# Visualizing chemical space networks with RDKit and NetworkX

**DOI:** 10.1186/s13321-022-00664-x

**Published:** 2022-12-28

**Authors:** Vincent F. Scalfani, Vishank D. Patel, Avery M. Fernandez

**Affiliations:** University Libraries, Rodgers Library for Science and Engineering, The University of Alabama, Tuscaloosa, AL 35487 USA

**Keywords:** Chemical space network, Chemical similarity network, CSN, Molecular similarity, Maximum common substructure, RDKit, NetworkX

## Abstract

This article demonstrates how to create Chemical Space Networks (CSNs) using a Python RDKit and NetworkX workflow. CSNs are a type of network visualization that depict compounds as nodes connected by edges, defined as a pairwise relationship such as a 2D fingerprint similarity value. A step by step approach is presented for creating two different CSNs in this manuscript, one based on RDKit 2D fingerprint Tanimoto similarity values, and another based on maximum common substructure similarity values. Several different CSN visualization features are included in the tutorial including methods to represent nodes with color based on bioactivity attribute value, edges with different line styles based on similarity value, as well as replacing the circle nodes with 2D structure depictions. Finally, some common network property and analysis calculations are presented including the clustering coefficient, degree assortativity, and modularity. All code is provided in the form of Jupyter Notebooks and is available on GitHub with a permissive BSD-3 open-source license: https://github.com/vfscalfani/CSN_tutorial

**Graphical Abstract:**

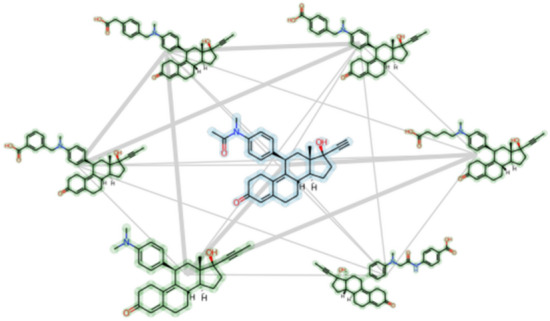

**Supplementary Information:**

The online version contains supplementary material available at 10.1186/s13321-022-00664-x.

## Introduction

Over the past decade, chemical space networks (CSNs) have been introduced as a way to visualize and interpret relationships within small molecule datasets. CSNs were designed as an alternative representation to coordinate-based visualizations using molecular descriptors. In a typical CSN, compounds are represented as nodes, and these nodes are connected with edges, where an edge is defined as some type of relationship between the compounds [1–3], including for example, 2D fingerprint-based Tanimoto similarity [4–7], substructure-based similarity [4, 5, 7, 8], or asymmetric Tversky similarity [9, 10].

In cases where the CSN edges represent a range of similarity values such as fingerprint-based Tanimoto similarity, the number of edges can be adjusted by incorporating a minimum threshold value; that is, only draw an edge if the selected relationship between the compounds are equal to or greater than the selected value. This is in contrast to, for example, in the case of matched molecular pair (MMP)-based CSNs, where the edges represent a binary relationship between the compounds (i.e., draw edge for MMP, no edge if not a MMP) [1, 2]. In CSNs, the molecular nodes can be visually represented as symbols such as circles or 2D chemical structure depictions, while edges are represented with lines. Additional layers of complexity can be added to the network visualization, for example, coloring the nodes by property value or changing the line style based on similarity value [1, 3].Vogt et al. note that CSNs are generally considered most useful to represent compound datasets on the order of 10 s to 1000 s of compounds, and that compounds within the dataset should have some level of similarity or other relationship that can be used to form connections within a network. In addition to the useful visualizations that CSNs provide, one advantage of generating a CSN is that well-established network science algorithms and statistical calculations can be applied in the subsequent analysis [2].

There are a variety of software and techniques reported in the literature used to create CSN visualizations [3]. Specific to small molecule CSNs, reported methods include in-house Java programs and Java Universal network/graph framework [5–7, 11], in-house Python or Java code and Gephi software [4, 8–10], and Python NetworkX and/or Cytoscape [12, 13]. Cheminformatics toolkits reportedly used for computing molecular relationships for use in CSNs include, for example, in-house implementations [9], Molecular Operating Environment [11], OEChem toolkit [7, 8, 10, 11, 13], and CDK [12]. An example has been reported of using RDKit in a chemical network workflow. Dunn et al. used a combination of RDKit and NetworkX to generate Chemical Library Networks, which are similar to CSNs, with the difference being that the nodes represent compound datasets, not individual compounds [14].

This article will demonstrate how to generate CSNs using RDKit [15], NetworkX [16], and Python. To our knowledge, no such tutorial exists in the published literature nor on the open web. In fact, we were not able to find any step by step code examples for how to generate CSNs regardless of the specific cheminformatics toolkit or other software used. We selected RDKit and NetworkX for this tutorial, in part because of our familiarly with these tools, but also because of the popularity of RDKit and Python in the cheminformatics community.

## Overview and organization of this manuscript

Our specific use-case for this manuscript is to create CSNs using RDKit and NetworkX with a dataset collected from ChEMBL associated with the glucocorticoid receptor (adapted from a dataset described by Zhang et al. [7]). We note that the primary focus of this manuscript is to demonstrate a CSN workflow from pairwise calculations to visualization, and not focus on identifying or hypothesizing any specific scientific conclusions from the sample glucocorticoid receptor data. Within the workflow, there are a variety of specific steps necessary including, for example, data curation, computing pairwise relationships of compounds, compiling data into the network data structure, and then plotting the network. Outlined below are the workflow steps we carried out. These workflow steps from loading data to analyzing basic network properties should not be taken as “*the workflow*”, but rather one workflow example of how to approach creating CSNs with RDKit and NetworkX.

Importantly, our methods were heavily influenced and adapted from the published reports by Bajorath and coworkers [1, 2, 5, 7]. From their reported methods, we were able to infer the necessary computations and steps for creating CSNs and turn these steps into Python code. While we used RDKit and NetworkX in this report, we have attempted to present the steps in a way where the methods and code could be adapted with other programming workflows. In fact, our original test implementation of CSN visualizations used Wolfram Mathematica, which we then re-wrote in Python code with RDKit and NetworkX.

For presentation and length considerations within this manuscript, we have focused on discussing selected key parts of the code for CSN calculations and CSN visualizations. The complete code and additional code comments are available as Jupyter Notebooks at https://github.com/vfscalfani/CSN_tutorial. The Jupyter Notebooks contain the same order of steps used in the manuscript. We recommend readers refer to both this manuscript and the complete code within the Jupyter Notebooks.

Code lines in the manuscript start with the iPython-like cell notation using the symbols, [«], denoting code input, and, [»], denoting code output. Integer values were not used as only selected pieces of the code are shown in this manuscript; that is, the code shown is not necessarily sequential. Lastly, in contrast to traditional scientific manuscripts with separate Methods and Discussion sections, we have combined the Methods with Discussion in an effort to create a readable and easy to follow tutorial style manuscript.

### Step 0—hardware, operating system, and conda environment setup

Ubuntu 18.04.6 LTS was used on an Intel-based laptop (Core i9-9980HK CPU @ 2.40 GHz × 16) with 32 GB of RAM. For the development environment, a conda (v4.14.0) python (3.10.6) environment was created with the following recipe in a terminal:


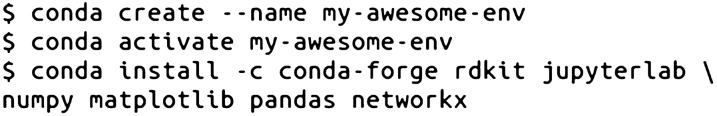


The specific versions installed were rdkit (2022.03.5), jupyterlab (3.4.7), numpy (1.23.3), matplotlib (3.5.3), pandas (1.4.4), and networkx (2.8.6).

### Step 1—ChEMBL data collection

Data was collected by browsing the ChEMBL web interface during September 2022. The data collection procedure was adapted from one of the datasets used by Zhang et al., specifically the glucocorticoid receptor antagonist dataset [7]. To compile a similar dataset, we first searched for "glucocorticoid" in the ChEMBL web interface and then applied several selections and filters in the following order:

1. Selected Targets
  Added Filters: Homo Sapiens
2. Selected CHEMBL2034 (Glucocorticoid receptor)
3. Selected Assays for Target CHEMBL2034
  Added Filters: Homo Sapiens, Single Protein Format, Scientific Literature, 9-Direct single protein target assigned, D-Direct protein target assigned
4. Selected Browse Activities
  Added Filters: Ki, molecular weight under 600, Target - CHEMBL2034
5. Exported and saved data as a CSV file, which contained 459 entries of compounds and associated ChEMBL data.

The exported CSV ChEMBL dataset is provided in the Additional file 1 and is redistributed according to the ChEMBL Data license with a Creative Commons Attribution-Share Alike 3.0 Unported License [17–19].

### Step 2—load the data into a Python variable and curate/prepare compound data

We used the Pandas data analysis library [20, 21] to read the glucocorticoid receptor data into a DataFrame variable, and then created a DataFrame with only the data we would be using in the CSNs, including the ChEMBL IDs, SMILES, and Ki values. The Pandas DataFrame variable was selected as it has a number of builtin methods that make data transformations straightforward on delimiter separated data. We applied several data cleanup procedures and checks to the collected glucocorticoid receptor data. First, compounds with no associated Ki values were removed. This was followed by checking for presence of salts as disconnected SMILES by basic string matching a period symbol. As string matching can miss edge-cases, it is preferred to fully parse the SMILES with a cheminformatics toolkit and then perform pattern matching with chemical perception. For example, one potential edge-case with disconnections is if any of the provided SMILES use dot disconnect bonds with ring closures; that is, the presence of a dot disconnect bond does not actually guarantee that the compound has more than one component [22]. As a result, we validated that all compounds in the glucocorticoid dataset did indeed contain only one fragment using the RDKit GetMolFrags function, which can be used to identify the number of molecule fragments:


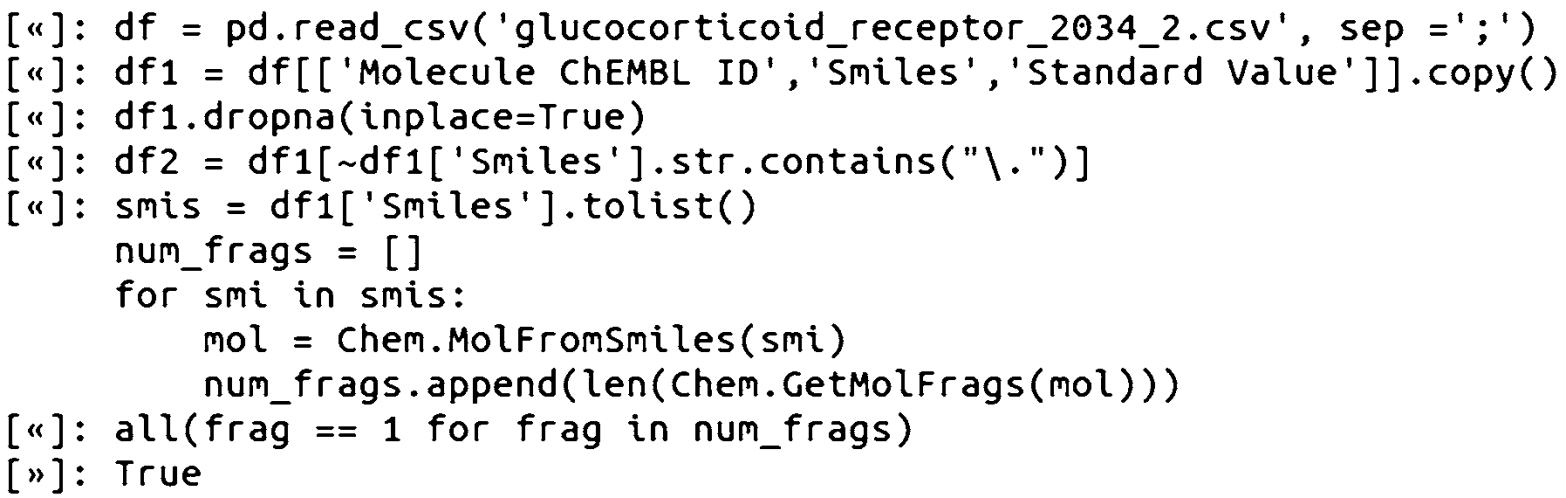


For datasets containing disconnected structures, there are several well-established procedures and tutorials in the cheminformatics literature for how to handle salts and other standardization protocols (vide infra). The next task was to merge duplicate compound data by averaging Ki values for identical compounds (equivalent ChEMBL ID and SMILES) with multiple Ki values [7]. The final filtered and merged list of the original ChEMBL provided SMILES, now totaling 404, were checked that they were unique from each other using Python sets. We also double checked this by parsing the ChEMBL SMILES and validating that the RDKit canonicalized SMILES were unique from each other:


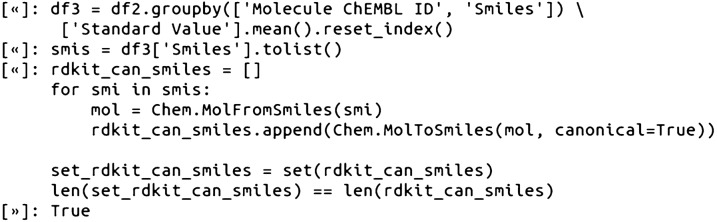


We did not perform any specific chemical structure standardization such as the normalization of functional groups, as we relied on the data and small molecule standardization protocols implemented in ChEMBL, for the purposes of this tutorial [23]. For more advanced discussions regarding the importance and methods of data curation in cheminformatics and structure standardization procedures, particularly when compiling data from multiple sources, see the published articles by Fourches et al.[24, 25]. In addition, the open-source presentation materials from the RSC Open Science Workshop from Landrum on molecular standardization, contain several examples with Python and RDKit [26].

### Step 3—Compile the network node data (compounds and associated pKi values)

For compiling the final node data, the Ki values in the pandas DataFrame were converted to pKi values (pKi = − log10(Ki)). This was followed by setting the DataFrame index column to the SMILES. In this case, setting the index column to SMILES was appropriate because we already validated that the SMILES strings were all unique from each other. The data was then saved as a dictionary variable, node_data, for all downstream calculations. In the node_data variable, SMILES are the unique dictionary keys, and the dictionary values are the associated ChEMBL ID and pKi data:


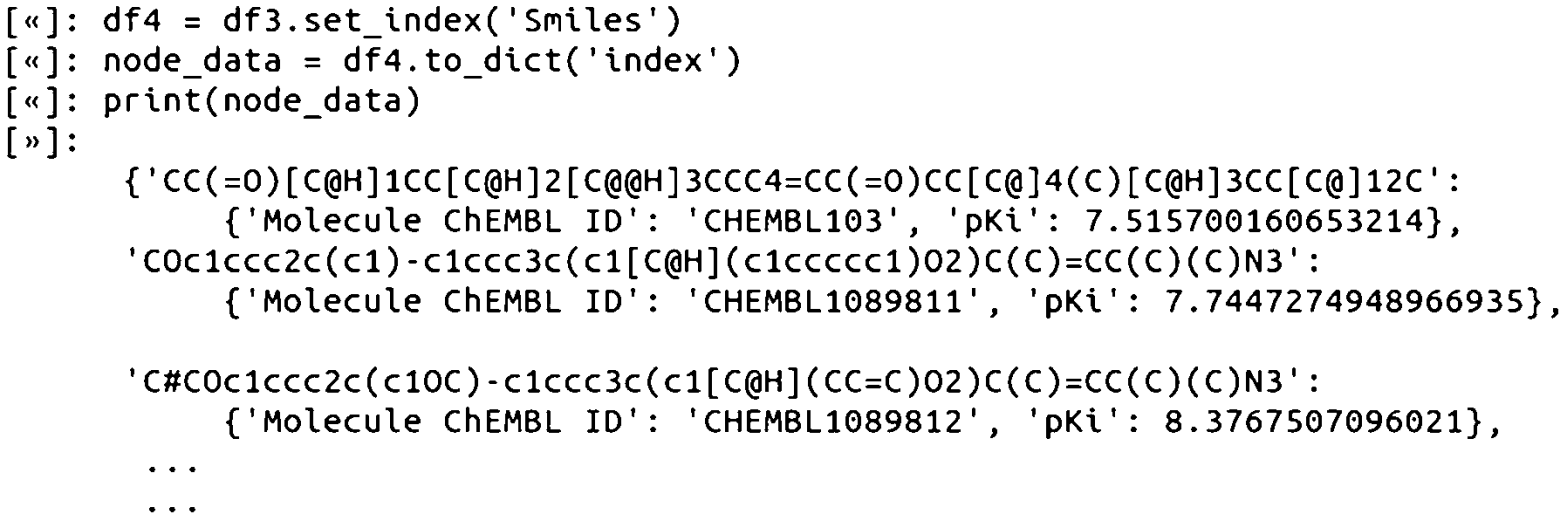


Using dictionary variables for both the node data and edge data (Step 4) was a convenient data structure for working with NetworkX and molecular data, as dictionaries are both straightforward to index and programmatically loop through.

### Step 4—compile network edge data by computing pairwise similarity relationships of compounds

We created two different edge datasets for the CSNs, one based on the Tanimoto Similarity of two compounds using RDKit fingerprints, and another CSN based on a maximum common substructure (MCS)-based similarity calculation [7]. To compute these similarity values, we first created a list of all possible unique combinations of the compounds using the Python itertools combinations method with the SMILES (reminder: the 404 final SMILES were previously checked for uniqueness from each other). The number of pair combinations, equivalent to possible edges, for 404 unique nodes was 81,406:


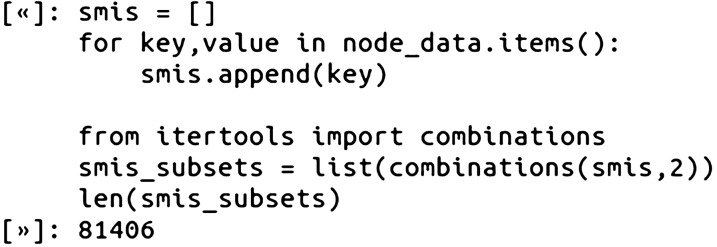


The number of pair combinations can quickly become difficult to handle computationally. For example, with 1000 nodes, the unique pair combinations would jump to nearly 500,000. Increasing the number of nodes to 2000 would create about 2 million pair combinations, several orders of magnitude greater than the number of nodes. In addition, as Zhang et al. notes, the MCS-based similarity calculation is computationally intensive, and so it is likely only reasonable to work with around 1000 nodes on standard personal computers, at least for MCS-based calculations [7].

The SMILES subset pairs were converted to a dictionary variable, named subsets, and the RDKit mol objects were added to the dictionary such that each subset pair has its own dictionary integer key and the associated SMILES and mol objects as the values:


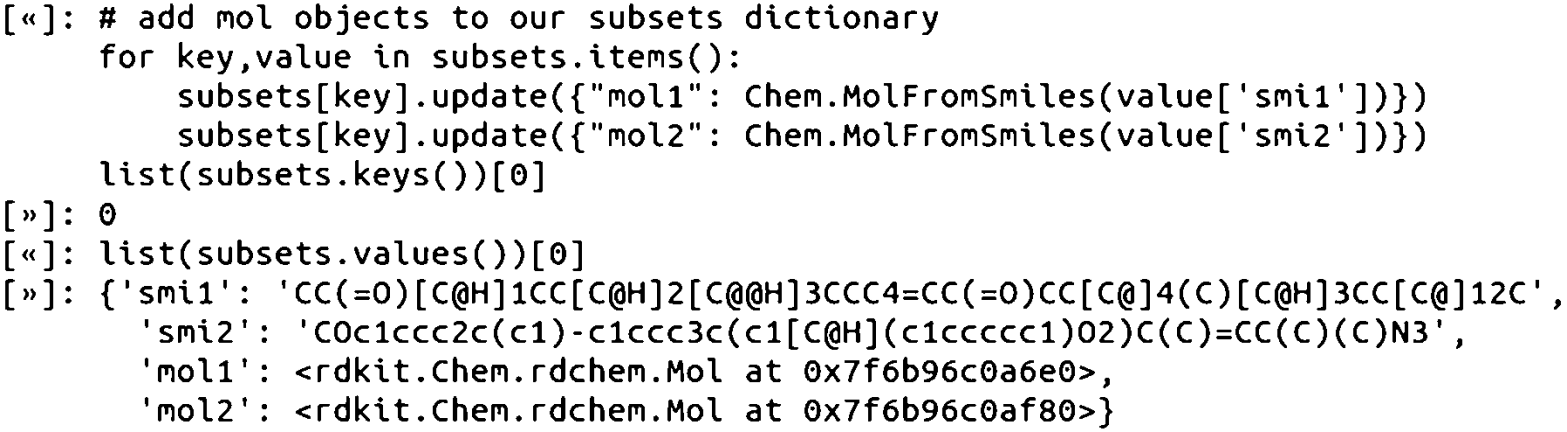


At this point, we computed the pairwise similarity values for the edges. The Tanimoto Similarity values were computed as follows using the RDKit fingerprints with default parameters:


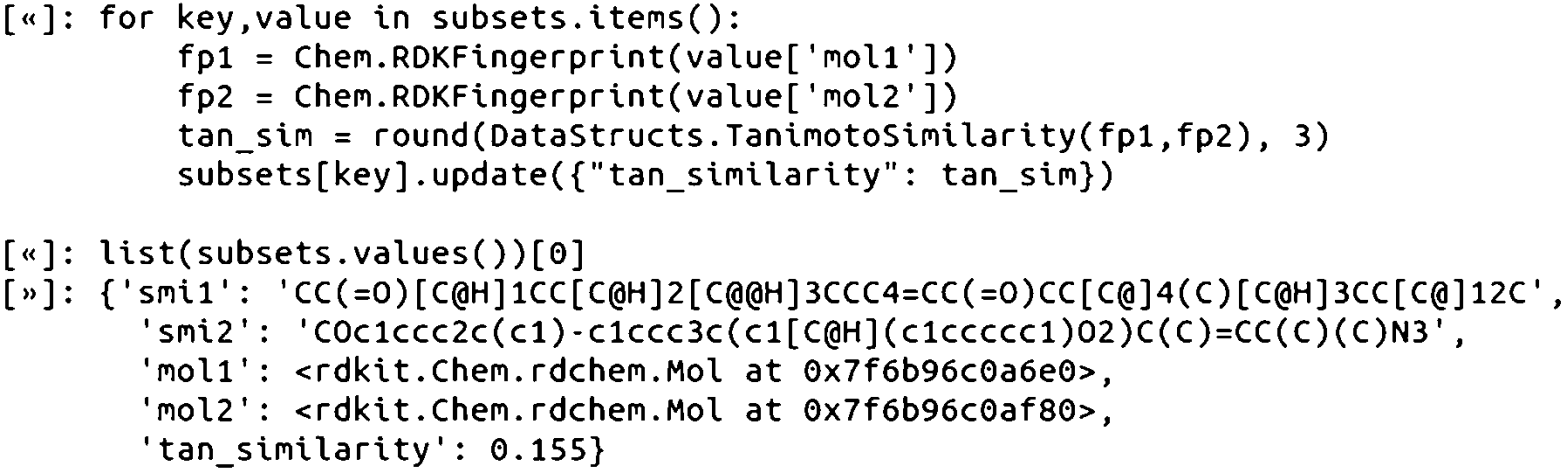


The RDKit fingerprint calculations for the ~ 81,000 pairs of compounds was fast, completing in about 3 min on the Core i9-based laptop used. Next, we wrote a Python RDKit function to compute the MCS-based similarity value using the equation reported by Zhang [7]. For the purposes of this tutorial, we added a 10 s timeout option to the RDKit FindMCS function, which serves to return the best MCS match found within 10 s, and speed up the calculations if certain comparisons are taking too long. This seemed like a reasonable parameter to add as well, as the RDKit “Getting Started” documentation suggests that most of the time, the FindMCS comparison takes less than a second [15].


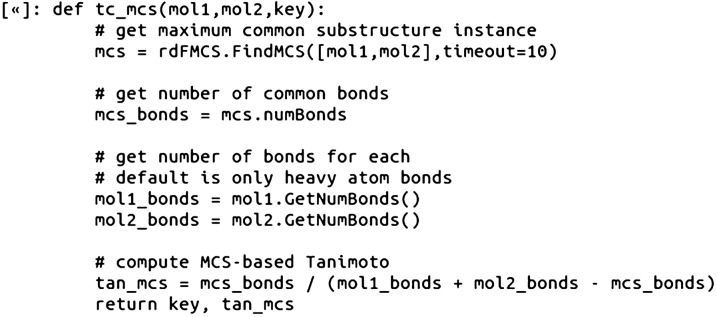


In addition to the FindMCS function being computationally demanding, Python defaults to single core processing. We found that computing 80,000 tc_mcs values was incredibly slow with single core processing. As a result, we used the Python multiprocessing module, which allowed us to loop through the 80,000 pairs of mol objects and compute the tc_mcs function across multiple processors (Jupyter Notebooks). With this method and using 14 of the 16 cores of an Intel Core i9-9980HK (2.4 GHz × 16) CPU, the computation took about one hour. The final subsets data structure was as follows:


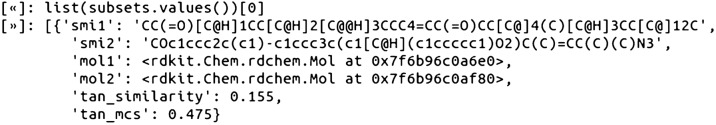


We recognize that not all readers will have access to a core i9 (or faster) computer workstation. As such, we ran the same computation on a 10 year old laptop with an Intel Core i5-2520 M (2.5 GHz × 4) CPU with 8 GB RAM, using 3 of the 4 cores and all ~ 80,000 MCS-based similarity values were computed in about four hours. Since the edge data computations can take a while to compute, these were saved as a Python pickle file, eliminating the need to re-compute the edge data for the downstream analysis and CSN visualizations (Additional file 1).

### Step 5—select a threshold value for edges to include in the network

The subsets dictionary variable, containing the edge data, was filtered based on the values of the Tanimoto similarity or the MCS-based similarity value; that is, not all possible edges are included in the CSNs. There are two approaches to filtering the edge data, the first would be to select a threshold value of similarity (e.g., > 0.5), and the second is to target a specific final edge density (edges / total edges) [7], then calculate the value of similarity needed to match the target edge density. We selected a filtering value of >= 0.68 for the RDKit fingerprint Tanimoto-based similarity CSNs, which created an edge density of 0.095 (~ 10%):


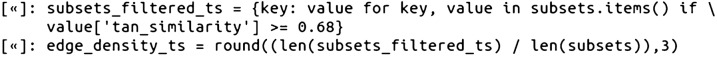


For the MCS-based similarity CSNs, the edge density of 0.095 was targeted to find the filtering value of 0.635 for the MCS-based similarity value (Jupyter Notebooks).

The selected edge density of ~ 10% is higher than values used in the literature for CSNs, which are typically 2–5% [2, 5, 6]. For the dataset we used, achieving an edge density of ~ 3% would require a threshold value of ~ 0.9 for the Tanimoto and MCS-based similarity CSNs. We selected a higher edge density for the purpose of this tutorial for two main reasons: (1) we wanted to incorporate a range of similarity values into the network visualizations in order to demonstrate creating different edge line styles based on these values, and (2) obtaining a lower edge density created a high number of small individual connected components in the graph, which we wanted to avoid to simplify the visualizations. Tuning and selecting an appropriate edge density is an important consideration when studying CSNs as many network properties are affected by edge density [2, 5–7]. Using the methods and code described in this article, it is straightforward to experiment with different edge densities by simply adjusting the threshold filtering value and executing the code for the downstream visualizations and calculations. Lastly, we removed any nodes (compounds) from the node_data dictionary variable that do not appear in the filtered edge data. This step removes the occurrence of nodes without any edges. For the tanimoto-based similarity CSNs the final number of nodes was 393 and 7739 edges, and for the MCS-based CSNs, the final number of nodes was 398 and number of edges was 7709.

### Step 6—add node and edge data to the network graph and plot

After initializing an empty graph variable, the edge data including the compound SMILES (smi1, smi2) and their associated similarity value (Tanimoto or MCS-based similarity) were added to the graph by looping through the filtered subset edge data created in Step 5:


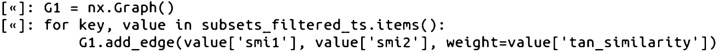


The NetworkX Graph.add_edge method automatically creates the nodes from the edges (nodes are the SMILES strings). However, we also added the node attributes using the Graph.add_node method in order to include the associated ChEMBL ID and pKi values:


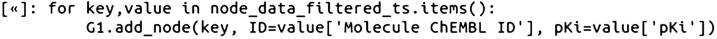


With the edge and node data added to the network graph, we now have all of the pieces in place to start creating visualizations. NetworkX provides graph drawing functionality using the Matplotlib library [27]. To create a basic plot, first a layout algorithm and parameters are selected, followed by the NetworkX draw function:


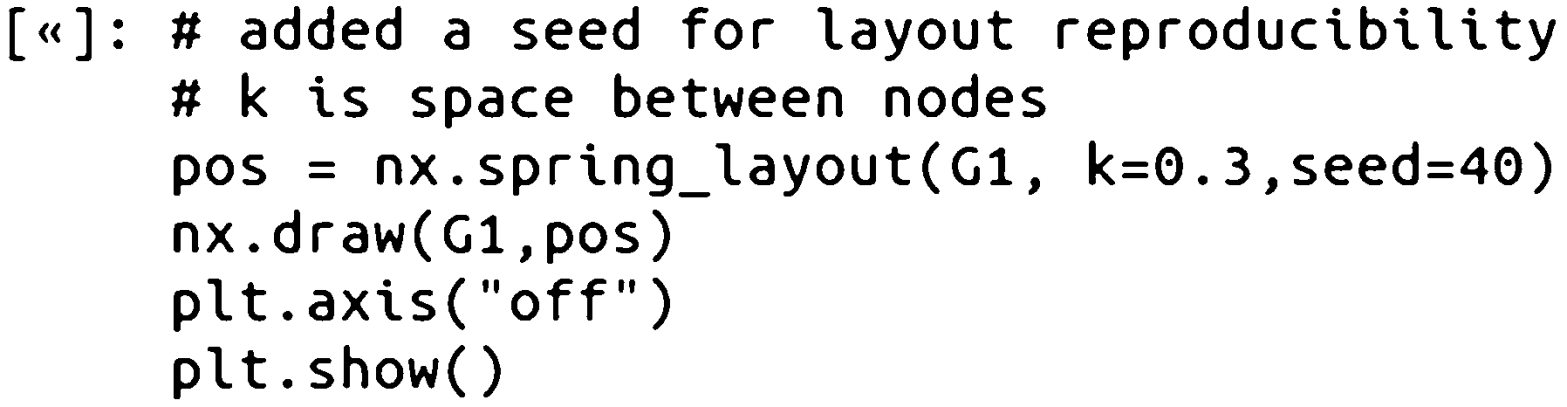


The spring 2D layout was selected because of its successful use in reported CSNs [5, 7, 9]. The NetworkX spring layout uses the Fruchterman-Reingold force-directed algorithm, which ultimately creates separate clusters/communities of connected nodes [7, 28]. By default, the NetworkX spring layout uses the weights of the edges (e.g., similarity values) within the force-directed calculations, where a larger weight equates to a stronger attractive force between the nodes in the algorithm. We found that the similarity weight values had minimal visual impact on the overall layout of the CSNs (perhaps due to their similar magnitude). As a result, it is likely that the edge distance between connected nodes and space between individual clusters of nodes do not scale with similarity relationship value, which is consistent with other applications of the Fruchterman-Reingold algorithm for CSNs without weighted edge values [2, 7].

### Step 7—adjust plot settings such as node color based on pKi value, node size, and add legends

**Fig. 1:**
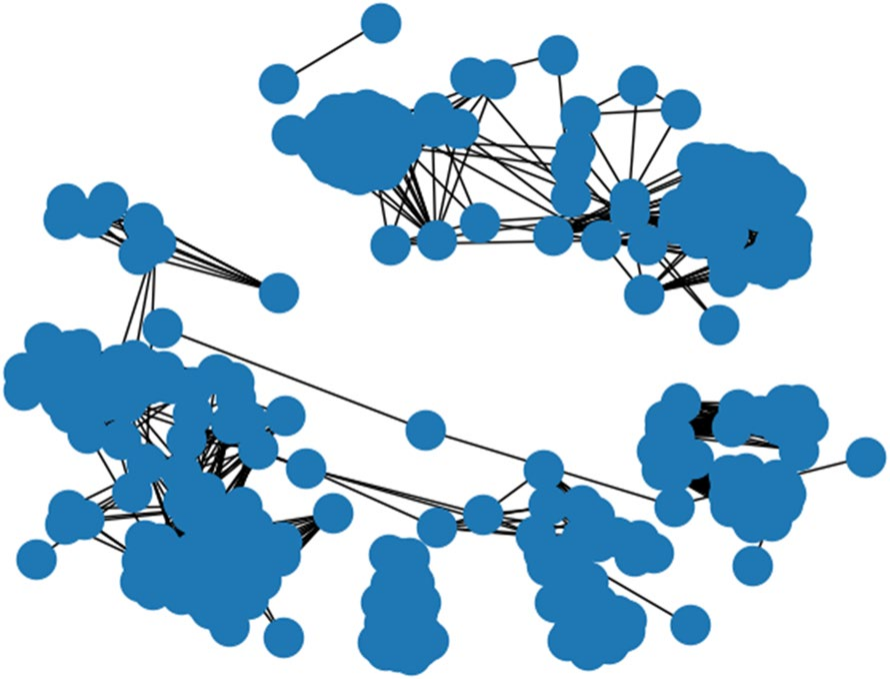
A basic spring layout CSN (Tanimoto Similarity variant) with the glucocorticoid dataset compounds. The Tanimoto Similiarity threshold was set to >= 0.68


Figure 1 is an acceptable basic CSN plot, however, by adjusting a few network drawing plotting parameters, it is possible to improve the amount of information that the CSN visualization conveys. The first adjustment to the CSN network drawing was to incorporate the pKi values as node colors. This was accomplished by looping through the graph’s node pKi attributes and creating a list of colors based on a conditional range of values from 4 to 11, with an increment of 1:

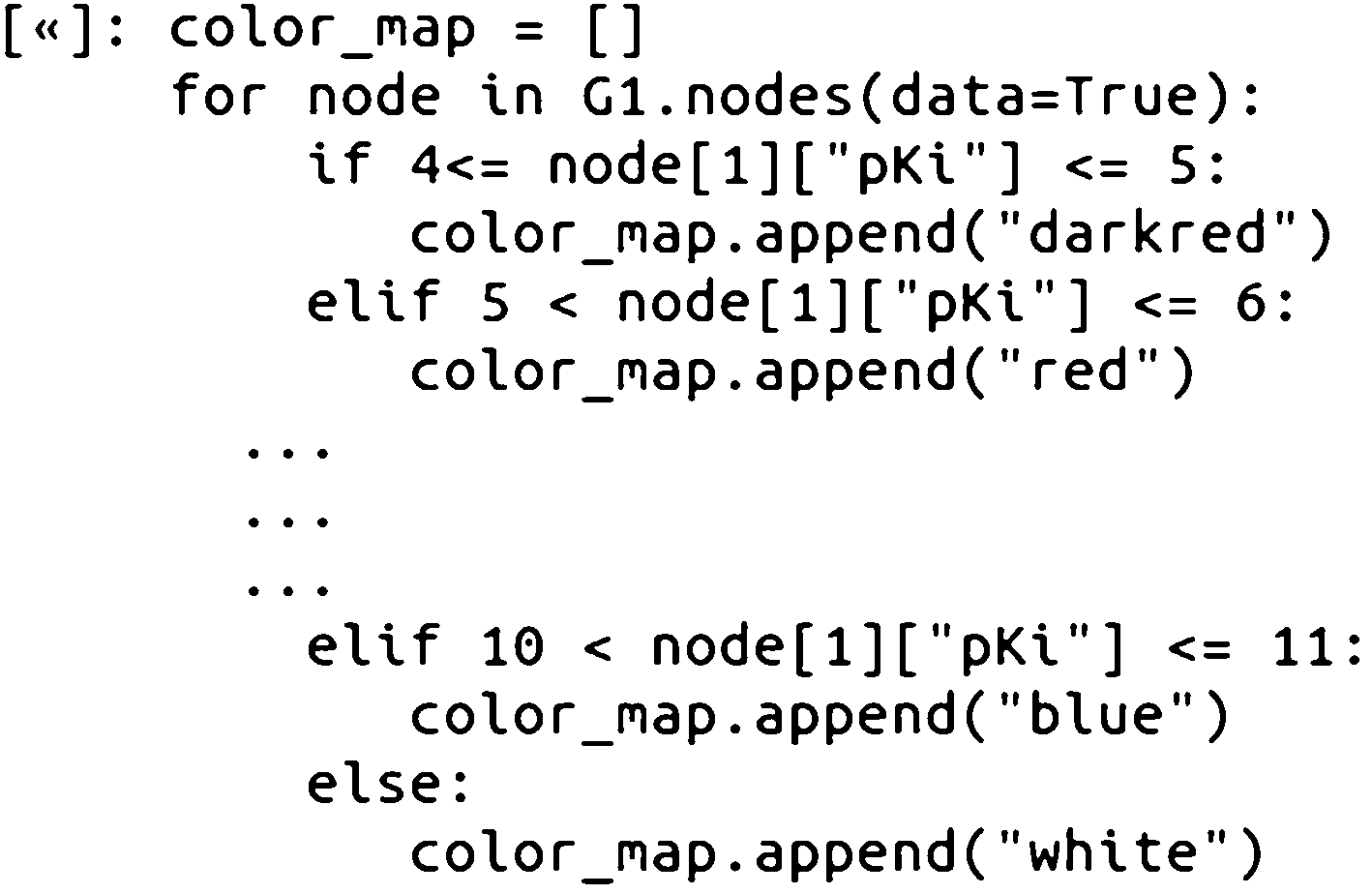


The color_map list can then be added into the network drawing with the node_color drawing option. Additional adjustments were also added in including a smaller node size, node edge border, and a grey color for the network edges:


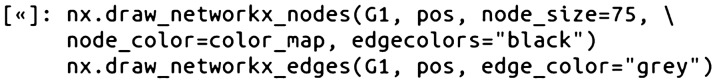


Since NetworkX uses Matplotlib for the drawing functionality, it is also possible to annotate the plot with a color bar legend by adding a second axis and defining the color bar bounds and attributes (Jupyter Notebooks).

### Step 8—Identify and plot specific components of the network

**Fig. 2:**
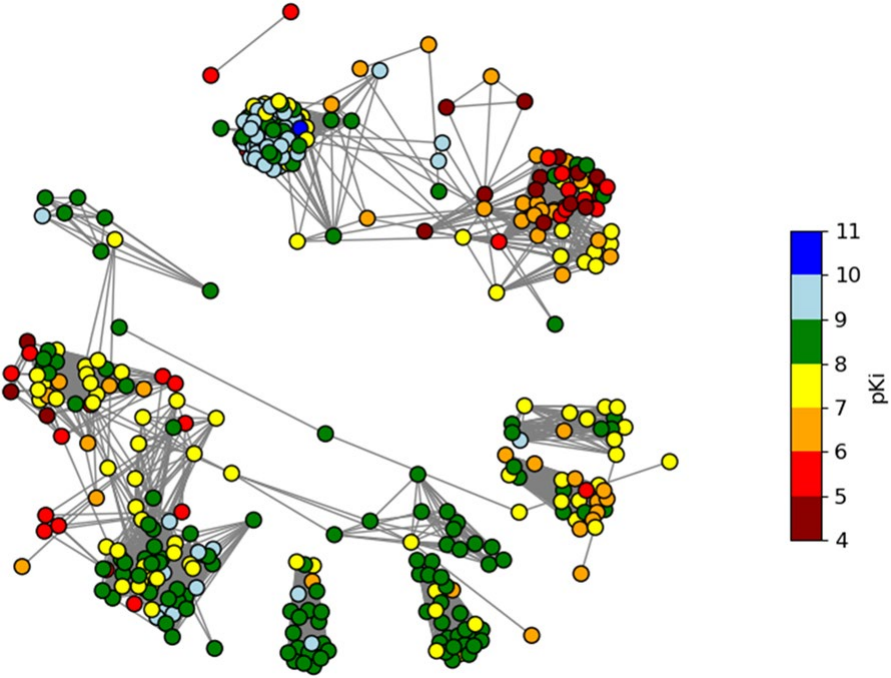
A spring layout CSN (Tanimoto Similarity variant) with the glucocorticoid dataset compounds. The Tanimoto Similiarity threshold was set to >= 0.68. Node color represents pKi value


Figures 1 and 2 depict the overall CSN visualization, however, it is useful to also identify and explore sections, or individual connected components of the graph. With NetworkX, the individual connected graphs can be enumerated and saved as a list:

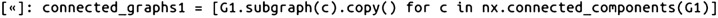


**Fig. 3:**
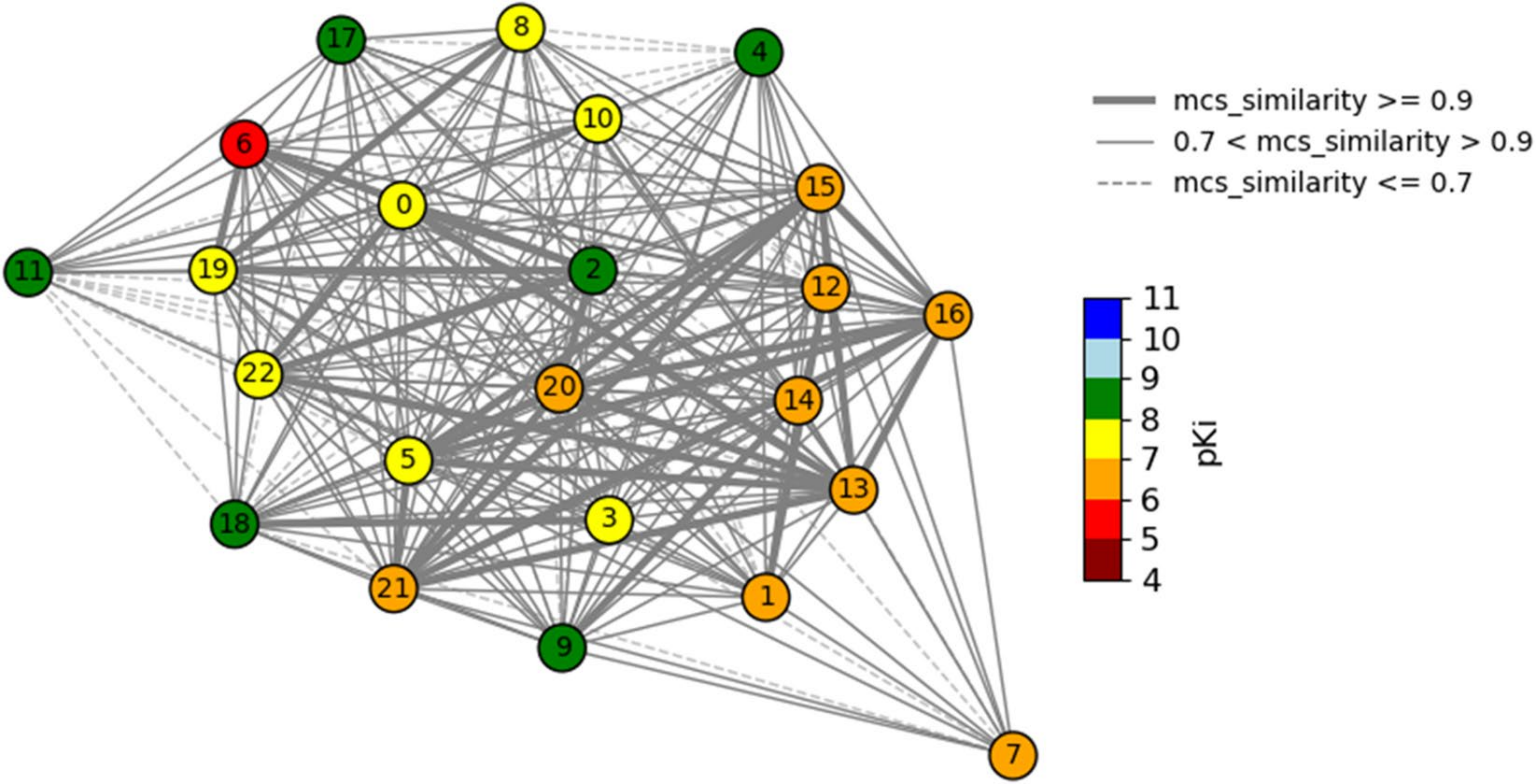
A spring layout CSN component (MCS Similarity variant) with the glucocorticoid dataset compounds. The nodes are labeled with index values, node color represents pKi value, and line style is dependent on MCS-based similarity value


The list of individual connected graphs can then be plotted similarly to the entire CSN network. However, since the connected graphs contain less nodes than the entire CSN network and offer a “zoomed in” view, it is possible to incorporate features in addition to node color such as node labels and different line styles based on similarity value [3, 29]. Node labels can be added by looping through the nodes and creating, for example an integer label for each node, while the line styles can be adjusted by filtering the edges into individual edge lists based on attribute value (Fig. 3):

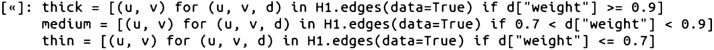


The custom node labels and edge lists can then be plotted with the NetWorkX drawing functionality using the draw_networkx_labels, draw_networkx_edges functions (Jupyter Notebooks).

Finally, the labeled nodes can be identified and further analyzed by looping over the graph nodes and indexing out the SMILES, ID values, and pKi values, into, for example, a DataFrame variable:


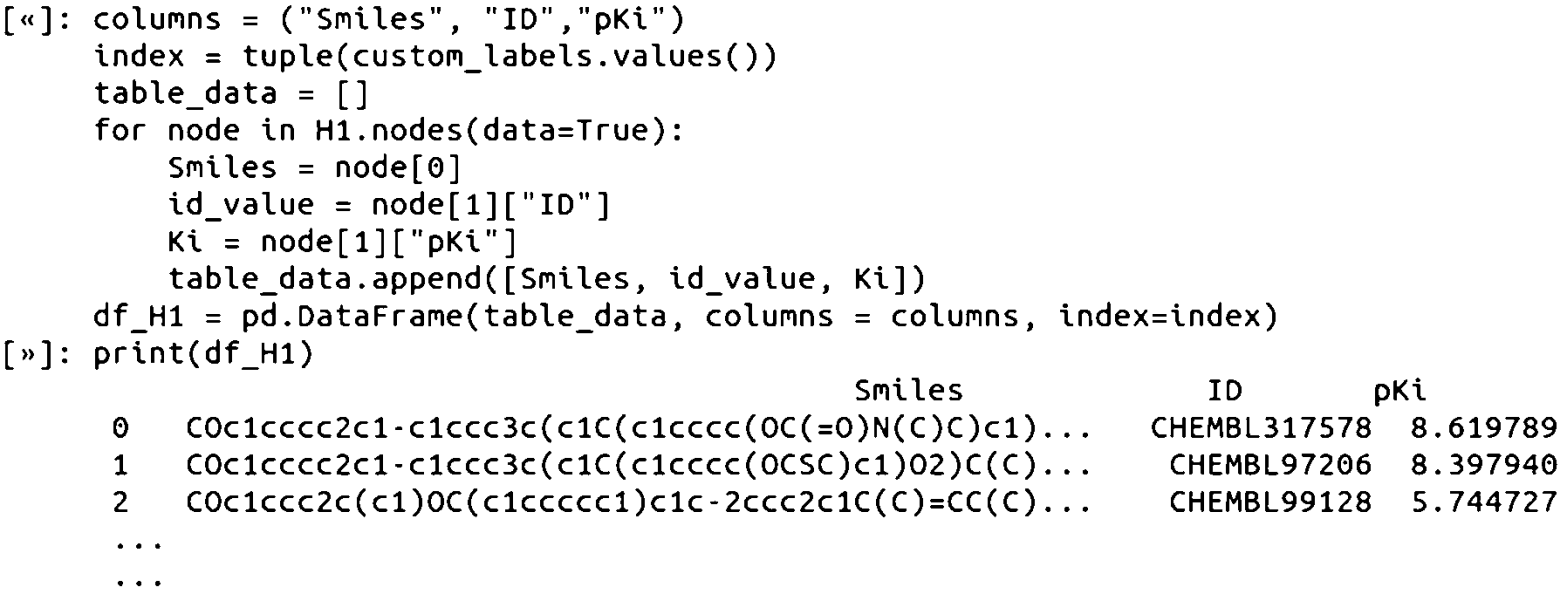


### Step 9—replace circle nodes with 2D chemical structures

**Fig. 4:**
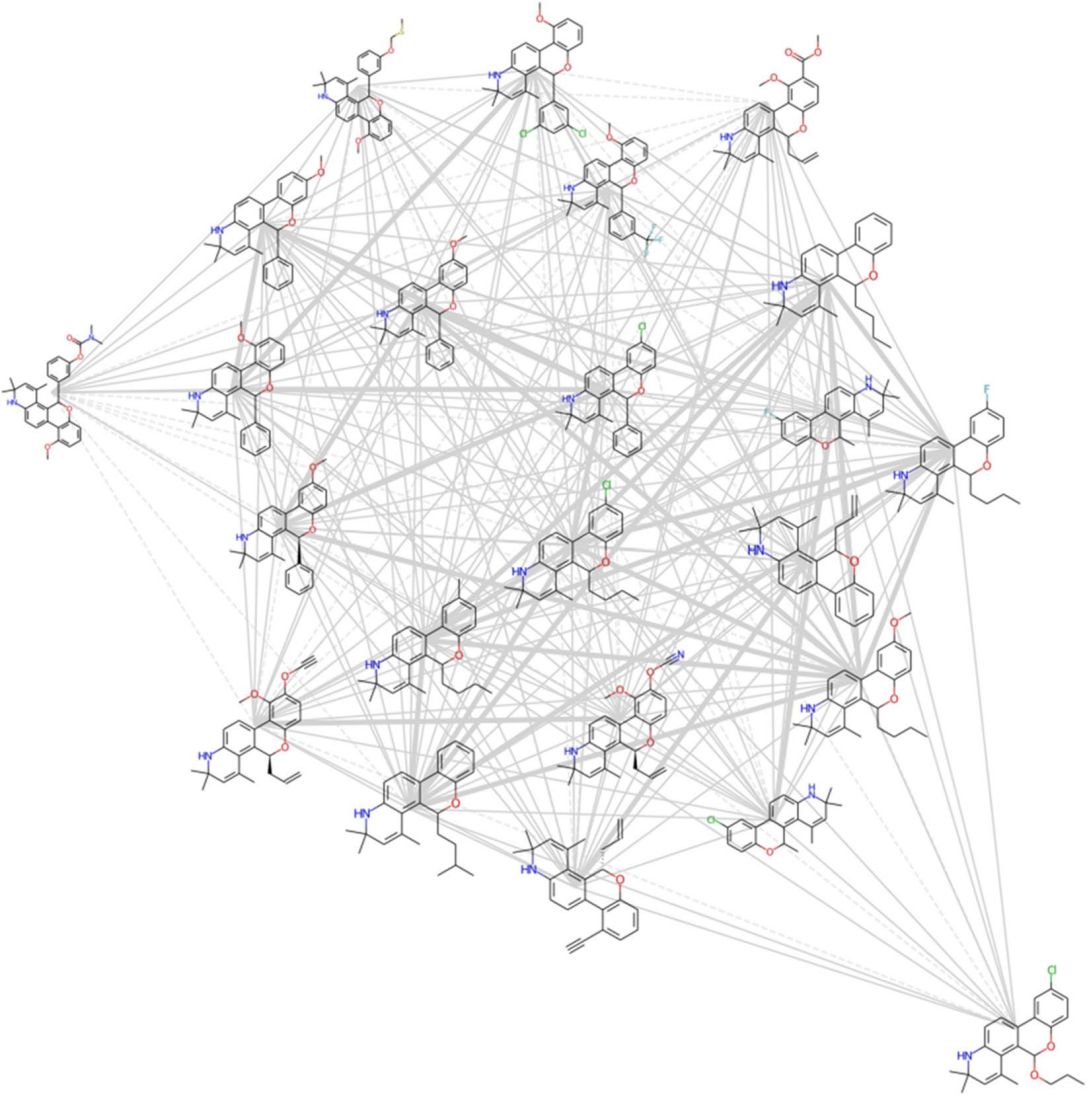
A spring layout CSN component (MCS Similarity variant) with the glucocorticoid dataset compounds. The nodes are plotted as 2D compound images and line style is dependent on MCS-based similarity value


As images can be plotted on Matplotlib axes and incorporated into network visualizations in NetworkX [30], it is possible to replace the representation of compounds as nodes with 2D structure depictions (Fig. 4). The workflow for plotting the nodes as 2D structures in a NetworkX graph included: (A) obtaining the positions to plot the structure images; (B) looping through the nodes (SMILES), and then; (C) using the RDKit rdMolDraw2D module to create 2D structure drawings as PNG images with transparent backgrounds. Step C of the workflow is shown below:

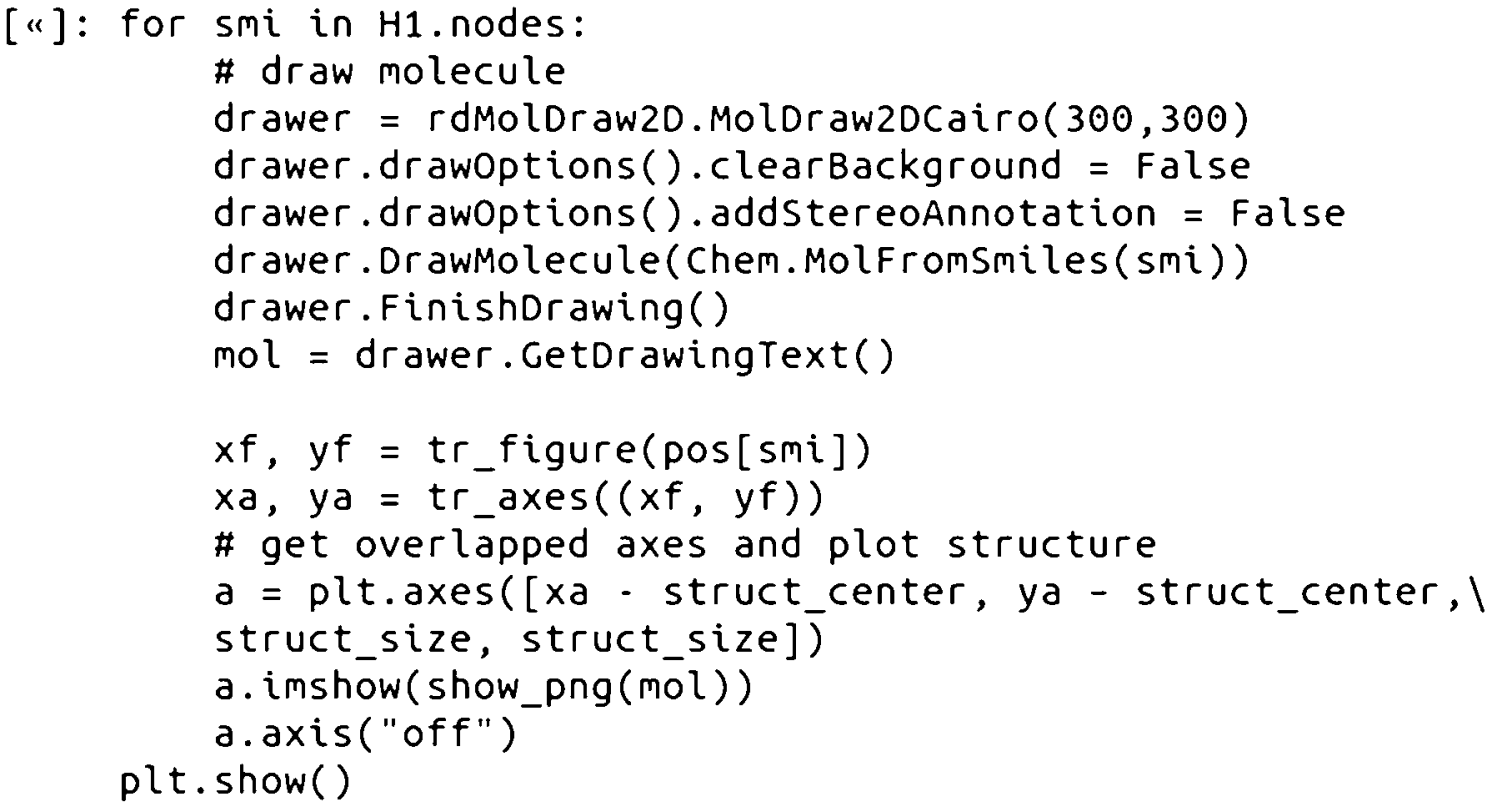


There are a couple limitations to be aware of when plotting nodes as 2D structures using this method. The first limitation is that the scaling size of the 2D structure images may need to be manually adjusted depending on the number of structures in the network (Jupyter Notebooks). This limitation is easily overcome, however, since it only requires adjusting one value manually until an appropriate size is found. We have found scaling values in the range of 0.04 to 0.1 to be appropriate for plotting 5–40 structure images.

One advantage of the Matplotlib/NetworkX CSN plotting method with 2D structure images is that the code to produce the network visualization is relatively straightforward and can be accomplished with about 15 lines of code. The produced static network images work well for creating high quality traditional scientific visualizations of networks on the order of 10 s of structures, however they are not well suited for attempting to navigate through an entire CSN or large component thereof (e.g., 100 s of nodes). For visualizing larger networks with 2D structure images, it is likely that some type of interactive network visualization would be needed. One method for creating interactive CSNs with 2D structures, which would incorporate well into the Python/NetWorkX/RDKit workflow described in this tutorial, would be to use the ipycytoscape Jupyter Notebook widget [31]. The ipycytoscape widget was recently featured in an RDKit User Group Meeting presentation for creating interactive scaffold networks (a type of fragmentation chemical network) [32, 33]. While creating interactive CSNs is beyond the scope of this tutorial, we plan to experiment with the ipycytoscape widget in the near future to evaluate its potential for creating CSNs with RDKit and NetworkX.

### Step 10—adjust 2D structure plot settings such as color based on pKi value and add legends

The basic 2D structure network depiction created in Step 9 (Fig. 4) can be enhanced by incorporating in color based on pKi value and adding in the ChEMBL IDs as labels. To accomplish this, we wrote an RDKit Python function, highlight_mol, that accepts SMILES, a string label, and a color name as input. The highlight_mol function then returns the depicted 2D drawing PNG image with all atoms and bonds highlighted:


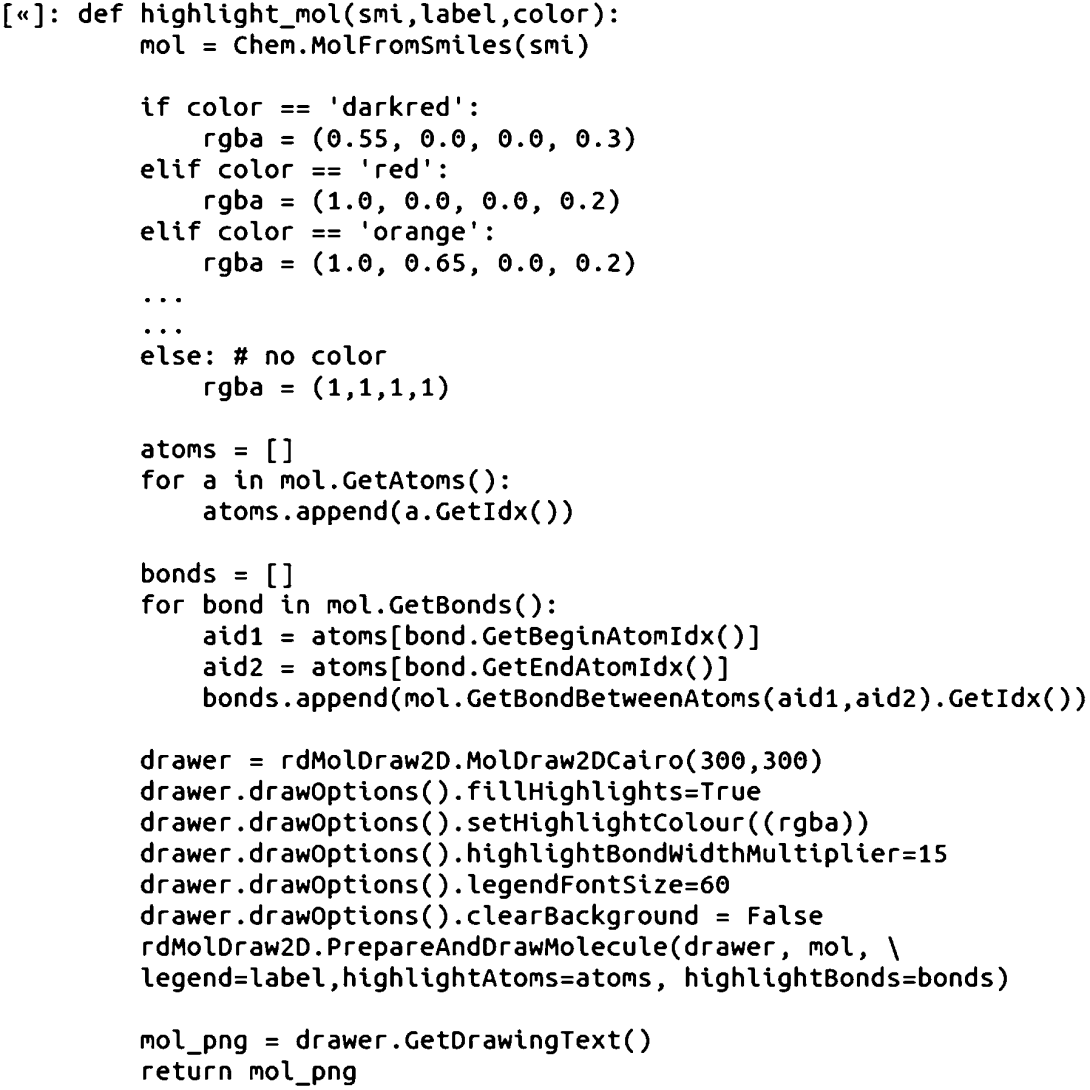


**Fig. 5:**
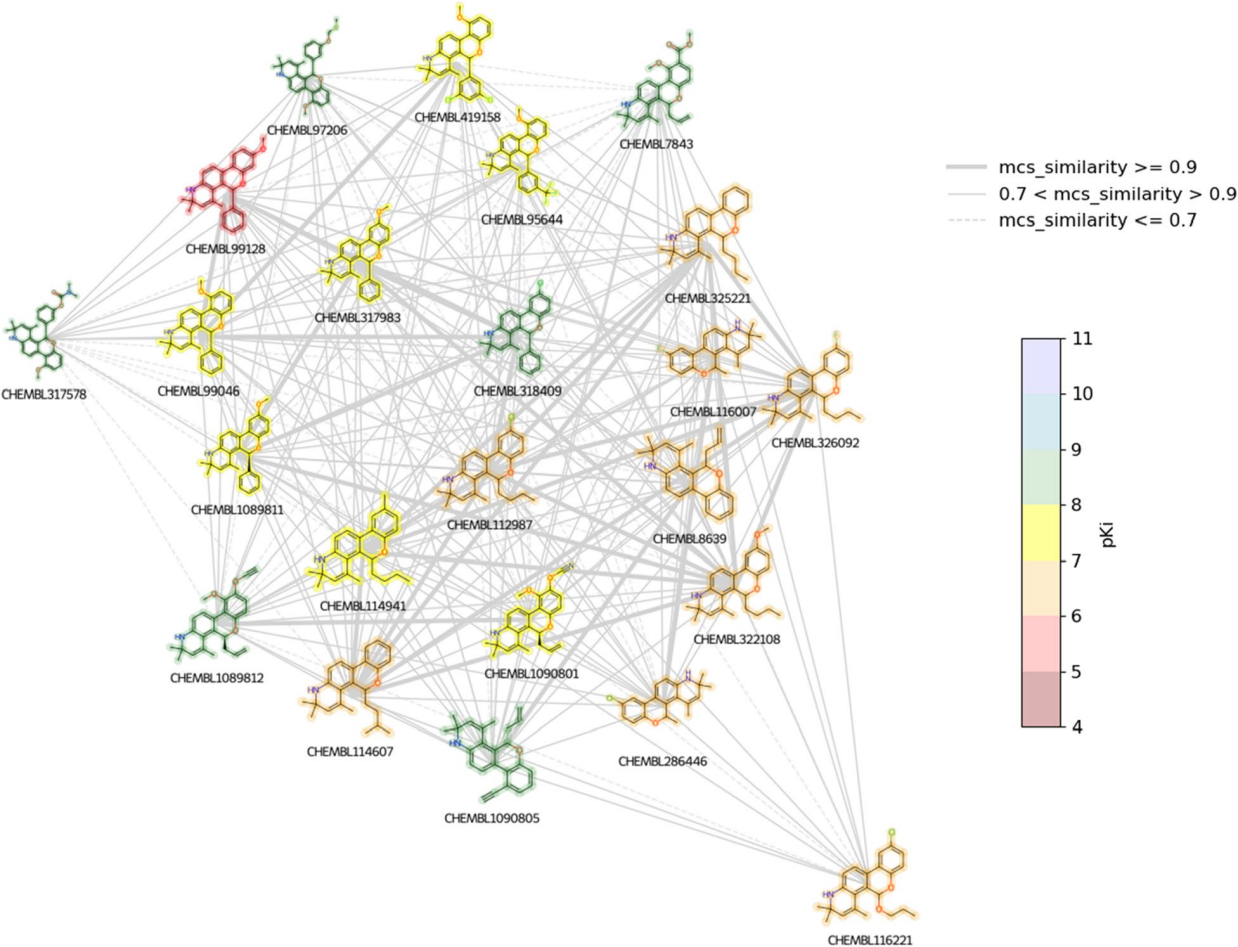
A spring layout CSN component (MCS Similarity variant) with the glucocorticoid dataset compounds. The nodes are plotted as 2D compound images with color highlighting for pKi values and line style dependent on MCS-based similarity value

The highlight_mol function was then incorporated into the workflow for plotting the nodes as 2D structures as described in step 9. The input data for the highlight_mol function was obtained by looping through each node in the graph and indexing out the respective pKi and ChEMBL ID value attributes (Jupyter Notebooks). To complete the visualization, a color bar and legend were added (Fig. 5).

### Step 11—compute network properties—clustering coefficient, degree assortativity, and modularity

NetWorkX includes many builtin functions for calculating network properties and applying network science algorithms into CSN analysis workflows. In this tutorial, we focused on demonstrating calculations for three common network properties including the clustering coefficient, degree assortativity, and modularity.

The clustering coefficient is a value between 0 and 1 that quantifies the ratio of the number of closed loops of three nodes that are in the network [34]. For example, in a CSN, for each pair of two compounds connected with an edge, the average clustering coefficient would then quantify the number of occurrences where the two compounds are both connected (similar) to a third neighboring compound [5, 7]. The clustering coefficient in NetworkX can be computed with the builtin clustering function using the CSN graph as input and optionally the weights of the edges. If the weights of the edges are used (e.g., similarity values), these are incorporated into the clustering coefficient calculation with a weight intensity factor [35, 36]. The average clustering coefficient for the weighted glucocorticoid CSN was 0.76 and 0.71 for the Tanimoto similarity and MCS-based similarity, respectively (Jupyter Notebooks).

The degree assortativity is a measure of correlation between the degrees of nodes (number of edges connected to node). The value ranges from -1 to 1. The larger the value, the more assortative the network, which, in short, indicates that similar node degrees are neighbors (e.g., high degree nodes connected to high degree nodes) [2, 5, 34]. CSNs reported in the literature typically have positive degree assortativity, which is a result of forming individual connected components or clusters of similar compounds in the network [5, 6]. In NetworkX, there is a builtin function, degree_assortativity_coefficient, that can be used with the CSN graph as input and optionally the similarity weights as input. The degree assortativity for the weighted glucocorticoid CSN was 0.96 and 0.84 for the Tanimoto similarity and MCS-based similarity, respectively (Jupyter Notebooks).

Modularity is a value that quantifies the separation of similar nodes (e.g. nodes connected with many edges) into distinct clusters or communities [5, 34]. To compute the modularity of the CSNs in NetworkX, first, communities need to be detected using an optimization function [6], followed by then inputting the detected community component graphs into the NetworkX modularity function. Using the NetworkX greedy_modularity_communities function for community detection of the weighted glucocorticoid CSNs, the Tanimoto similarity network had 18 communities with a modularity value of 0.62, and the MCS-based CSN had 13 communities with a modularity of 0.71 (Jupyter Notebooks).

**Fig. 6:**
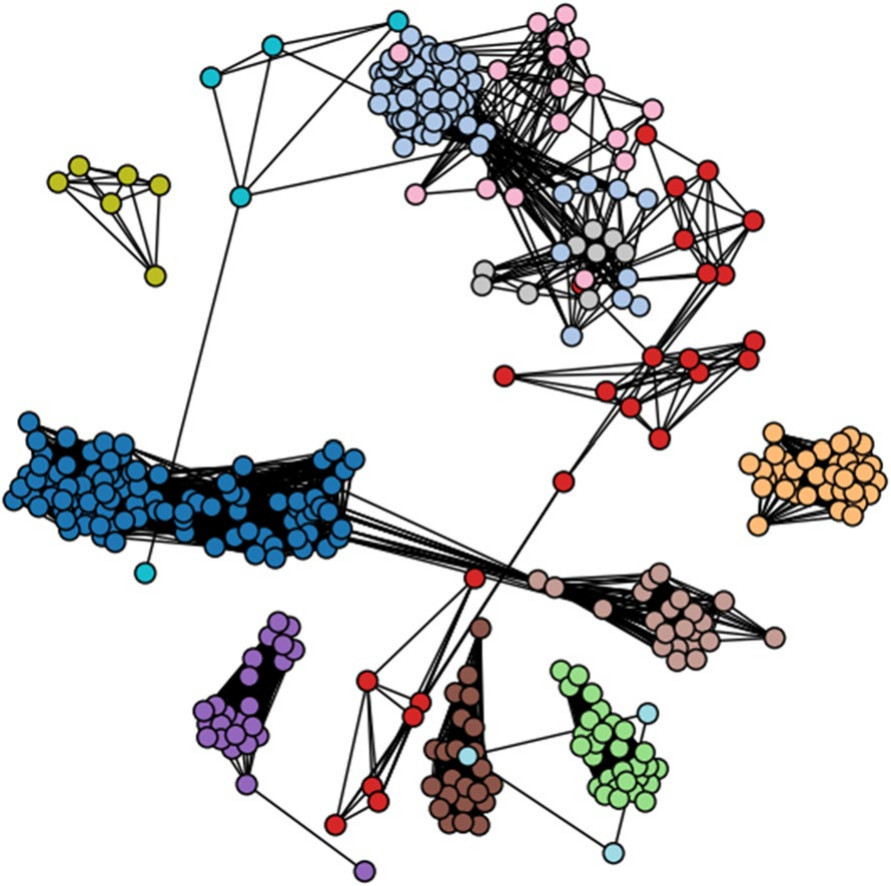
A spring layout CSN (MCS Similarity variant) with the glucocorticoid dataset compounds. The nodes are colored based on community cluster detected using the NetworkX greedy_modularity_communities function


Since the NetworkX community detection functions allow saving a list of the individual communities as nodes, it is possible to then loop through the list of nodes and add distinct colors to each node depending on the community for the CSN visualization (Fig. 6):

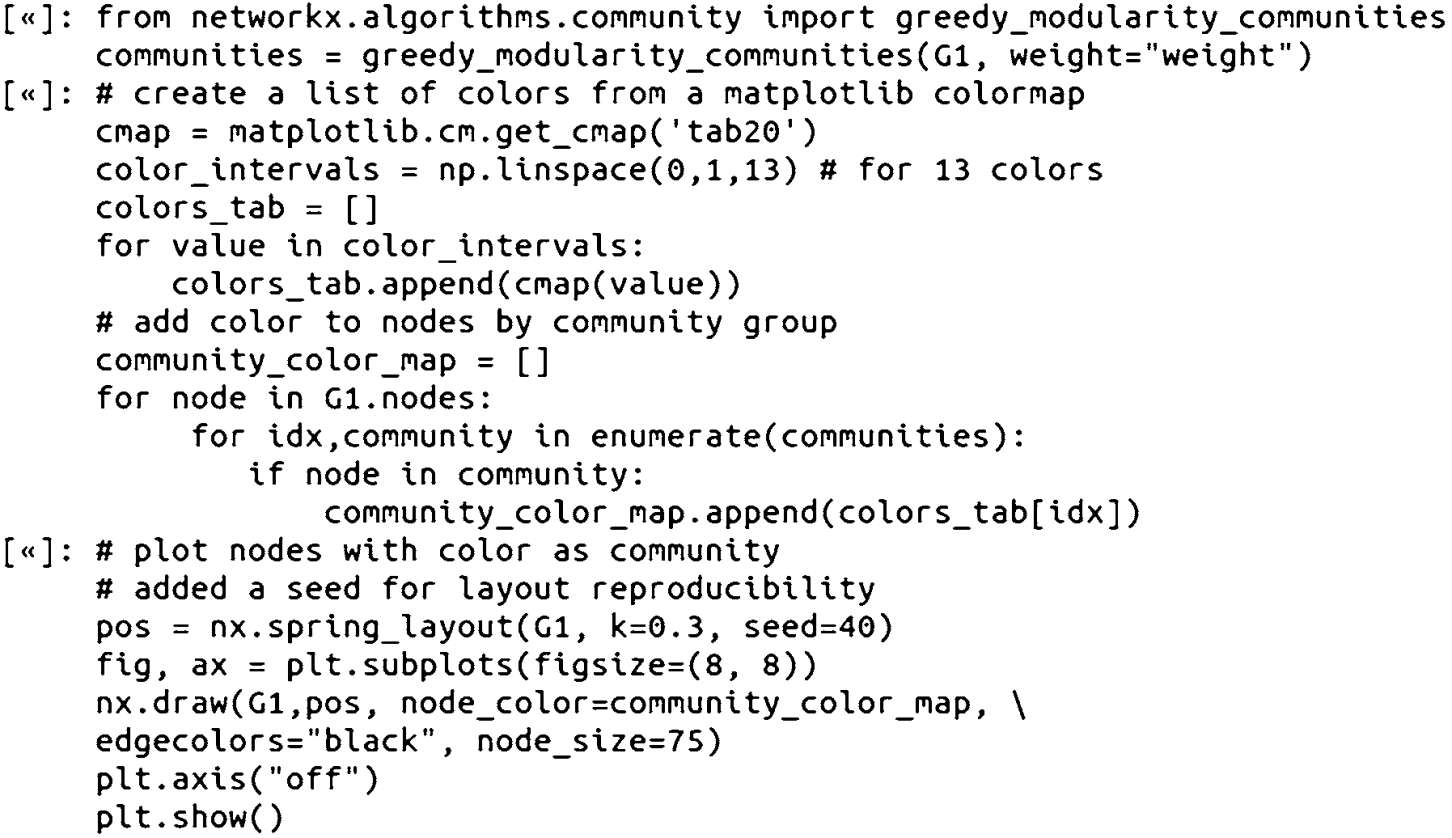


Interestingly, while we included the similarity edge values as an optional weight parameter in the spring layout, clustering coefficient, degree assortativity, and community detection network calculations, CSNs in the literature do not generally include the similarity weights into the network. Studying weighted CSNs is an area of interest and opportunity as pointed out in a 2016 review article by Vogt et. al [2]. However, to our knowledge there has only been a single report studying weighted CSNs. Ito and Ohnishi created weighted CSNs based on Tanimoto similarity relationships without incorporating any minimum threshold to include edges into the network; that is, all edges are included in the network, and the topology of the network is not influenced by a minimum similarity value [37]. As such, there is certainly an opportunity for the cheminformatics community to further evaluate the suitability of weighted CSNs and if a combination of a threshold parameter and weighted edges (as in this tutorial) would afford new information in the analysis of CSNs.

## Conclusions

This tutorial demonstrated how to use RDKit and NetworkX to create CSNs based on RDKit fingerprint Tanimoto similarity and a maximum common substructure-based similarity. A variety of CSN visualizations of varying complexity were created including CSNs with circle node representations, node color based on bioactivity attribute, edge thickness dependent on similarity value, and 2D structure depictions as nodes. With this manuscript and the included Jupyter Notebooks (Additional file 1), readers interested in generating CSNs should be able to adapt the code for their own use in research and teaching. We hope that code in this tutorial will provide a starting point for more CSN related cheminformatics research, including further comparison of CSNs to weighted CSNs (incorporating edge values into network calculations), which, to our knowledge has not yet been extensively studied in cheminformatics [2]. In addition, we suspect that selected parts of the tutorial workflow described in this report could be adapted for generating chemical library networks (where nodes represent collections of compounds) [14]. Finally, we plan to continue our work on CSN tutorials by creating interactive CSNs in the near future.

## Supplementary Information

**Additional file 1.** ChEMBL glucocorticoid dataset (CSV file).

## Data Availability

All code (as Jupyter Notebooks) needed to reproduce the results in this article are available on GitHub with a BSD-3 License: https://github.com/vfscalfani/CSN_tutorial

## Abbreviations

CSN: Chemical space network
MMP: Matched molecular pair
MCS: Maximum common substructure
